# Structural differences between non-lucid dreams, lucid dreams and out-of-body experience reports assessed by graph analysis

**DOI:** 10.1038/s41598-023-46817-2

**Published:** 2023-11-09

**Authors:** Francisco T. Gallo, Ignacio Spiousas, Nerea L. Herrero, Daniela Godoy, Antonela Tommasel, Miguel Gasca-Rolin, Rodrigo Ramele, Pablo M. Gleiser, Cecilia Forcato

**Affiliations:** 1 Laboratorio de Sueño y Memoria, Departamento de Ciencias de la Vida, Instituto Tecnológico de Buenos Aires (ITBA), Iguazú 341, (1437) Capital Federal, Buenos Aires, Argentina; 2 Consejo Nacional de Investigaciones Científicas y Tecnológicas (CONICET), Godoy Cruz 2290, (1425) Capital Federal, Buenos Aires, Argentina; 3 Sensorimotor Dynamics Lab (LDSM), Universidad Nacional de Quilmes, Bernal, Argentina; 4 Laboratorio Interdisciplinario del Tiempo y la Experiencia (LITERA), Universidad de San Andrés, Victoria, Argentina; 5 Instituto Superior de Ingeniería de Software Tandil (CONICET/UNCPBA), Tandil, Bs. As. Argentina; 6 Asociación Internacional de Onironautas, Carmelo Betore Bergua 2 Casa 6 9C, 50014 Zaragoza, Spain; 7 Centro de Inteligencia Computacional, Instituto Tecnológico de Buenos Aires (ITBA), Iguazú 341, (1437) Capital Federal, Buenos Aires, Argentina; 8 Laboratorio de Neurociencia de Sistemas Complejos, Departamento de Ciencias de la Vida, Instituto Tecnológico de Buenos Aires (ITBA), Iguazú 341, (1437) Capital Federal, Buenos Aires, Argentina

**Keywords:** Neuroscience, Psychology

## Abstract

Dreaming is a complex phenomenon that occurs during sleep, involving various conscious dream experiences. Lucid dreams (LDs) involve heightened awareness within the dream environment, while out-of-body experiences (OBEs) involve the sensation of being outside one’s physical body. OBEs occur during sleep paralysis (SP), where voluntary movements are inhibited during sleep/wake transitions while remaining aware of the surroundings. The relationship between LDs and OBEs is debated, with some viewing them as distinct phenomena and others considering them different manifestations of the same underlying experience. This study aimed to characterize non-lucid dreams, LDs, and OBEs by analyzing dream reports’ structural properties. OBE reports displayed a condensed and interconnected network structure compared to non-lucid dreams and LDs. Additionally, OBE reports exhibited a specialized network structure, with specific nodes playing a more central role. These findings suggest that OBE dreams may have a more coherent and unified narrative, with certain nodes being pivotal in the network structure.

## Introduction

Dreaming is a complex and intriguing phenomenon that occurs during sleep^1^. However, studying and understanding dreams presents numerous challenges due to its subjective nature and the inherent difficulty in directly observing conscious experiences. To understand the nature of dreaming, researchers must rely on the retrospective reports of individuals after they have awakened^2^. Among the various forms of dreams, two types of conscious dream experiences have received particular attention: lucid dreams (LDs) and out-of-body experiences (OBEs). LDs are characterized by heightened awareness within the dream environment^3,4^ and can occur during rapid eye movement (REM) sleep, but they have also been observed during sleep onset (N1) and light sleep (N2) stages^5–7^. OBEs involve the sensation of being outside one’s physical body and observing the world from this outside perspective^8–14^. They occur during sleep paralysis (SP)^13^, which is characterized by immobility while remaining aware of the surroundings^15^, but they can also occur during wakefulness^16,17^.

The relationship between LDs and OBEs, which occur during sleep, remains a topic of ambiguity within the existing literature. Different authors have presented contrasting viewpoints, resulting in varying opinions regarding their classification and distinctiveness. Some researchers argue that OBEs should be classified as a subtype of LD^18–21^, highlighting their shared characteristics and overlapping features. These include a heightened level of awareness within the dream state, enabling individuals to consciously perceive and interact with the dream environment. On the other hand, other researchers view OBEs as a related yet distinct phenomenon from LDs^3,22^.

As a result, the identification of specific electrophysiological signatures would be necessary to differentiate these experiences. LDs have been associated with an increase in low-gamma oscillatory activity (40 Hz) in the frontal and temporal regions of the brain^23^. However, the specific sleep stage and predominant EEG oscillations associated with OBEs are still unknown. Nevertheless, considering that OBEs occur during SP, which takes place during sleep/wake transitions^24^ and involves mixed alpha and theta brain waves^22^, it suggests the involvement of different predominant brain oscillations. In 2017, Siclari et al. showed that variations in brain oscillations during sleep can lead to perceiving the presence or absence of dream content^25^. The authors found that reports of dream experience were associated with local decreases in low-frequency activity and increases in high-frequency activity in posterior cortical regions, as observed through high-density electroencephalography recordings. Building upon these findings, it is to be expected that differences in brain activity patterns during LDs and SP are reflected in the dream content reported in LDs and OBEs.

There are at least two types of analyses used to study dream reports (extensively reviewed in Ref.^26^). One of them includes the analyses of the content^27^ and the other involves graph theory analysis^28^. The latter represents speech as a graph and computes mathematical qualities to quantify local and global topological characteristics of the reports. Graph analysis has been shown to be effective in differentiating dream reports in patients with psychosis such as schizophrenia and bipolar disorder^28,29^. This underscores the potential of graph-based methodologies to capture variations in dream reports beyond semantic content. When applied to the comparison between dreams during REM and dreams during NREM sleep^30^ in healthy participants, graph analysis has revealed quantitative differences that complement the previously identified qualitative disparities in phenomenology (see^30^). Essentially, dream reports stemming from different sleep phases exhibit structural differences in graphs that reflect distinct patterns of brain activation, thus supporting the feasibility of utilizing graph analysis to discern subtle variations in dream experiences. It has proven effective in distinguishing between dream reports from individuals with varying narrative capabilities, as well as between dream reports from different sleep phases in the general population. Unlike content analysis, which primarily relies on differences in semantic content, graph analysis has the potential to examine how OBEs differ from other types of dreams and enables us to capture the structural organization of dream reports, which goes beyond the mere analysis of textual content. Since the classification of which narratives are lucid and which are OBE is based on a classification extracted from their content, such as bodily sensations, the feeling of “leaving” the body, the realization that one is dreaming, etc., analyzing the content itself could introduce biases stemming from the classification process. In contrast, when analyzing the reports using graph theory, one can dissociate from the specific content, making the analysis objective and quantitative.

Here we performed an exploratory analysis of the word-by-word structural organization using graph theory to compare the structure of reported experiences to better understand the differences between non-LDs, LDs and OBEs from SP. For that, dream reports were collected from 60 individuals and divided into three groups based on their history of lucid dreaming and OBE: non-lucid dreamers, who had never experienced either LDs or OBEs; lucid dreamers, who had experienced LDs but not OBEs; and OBE dreamers, who had experienced both LDs and OBEs. The dataset included 916 reports (728 non-lucid dreams, 122 LDs, 68 OBEs). The reports were presented as directed graphs, with words serving as nodes and consecutive words connected by a directed, unweighted edge.

## Materials and methods

This study presents an analysis of a dataset previously collected by our research team at the Sleep and Memory Lab from the Instituto Tecnológico de Buenos Aires (ITBA)^31^. We obtained informed consent from all subjects prior to their participation in the study and provided all participants with a written explanation of the study procedures and their right to withdraw at any time without penalty. This study was approved by the Biomedical Research Ethics Committee of Alberto C. Taquini Institute for Translational Medicine Research (IATIMET), in accordance with the principles expressed in the Declaration of Helsinki.

### Dream journal and classification

In the original data collection effort, participants recorded their dreams for 2 months, noting the time, date, level of awareness, and description of each dream, as well as how they became lucid (if applicable). Two independent taggers classified dreams based on descriptions provided in a journal. A dream was considered lucid if the dreamer was either directly or indirectly aware. An OBE was identified if the dreamer described leaving the body or reported an aura (reported in Ref.^13^). We discarded 14 vague or unspecific reports, resulting in a sample of 916 dreams (731 non-LDs, 117 LDs, and 68 OBE dreams).

### Text processing and analysis

We processed the text of dream reports using Natural Language Processing (NLP) techniques to prepare it for sentiment and conceptual analysis. The spaCy library (https://spacy.io/) with pre-trained language models for Spanish was used for language processing. Tokenization was performed to split the text into meaningful elements called tokens or words. We then carried out part-of-speech (POS) tagging to mark the words in the text as corresponding to a specific part of speech (e.g., noun, verb, or adjective) based on their context in a given sentence. The final step of this process was lemmatization, which groups different inflected forms of words into a single element, known as the lemma or dictionary form. This way, words with the same lemma could be analyzed together as a single concept despite their different inflections or derivations of meaning. After performing text processing, we built a graph for each dream considering the entire text of the dream report. The nodes in the dream graphs were lemmas of the original words, and edges were established for words occurring consecutively in the text. Only lemmas corresponding to words with the POS tags NOUN, VERB, and ADJ were included in the graph construction, as these words contribute to content description.

### Speech graph attributes

**Figure 1:**
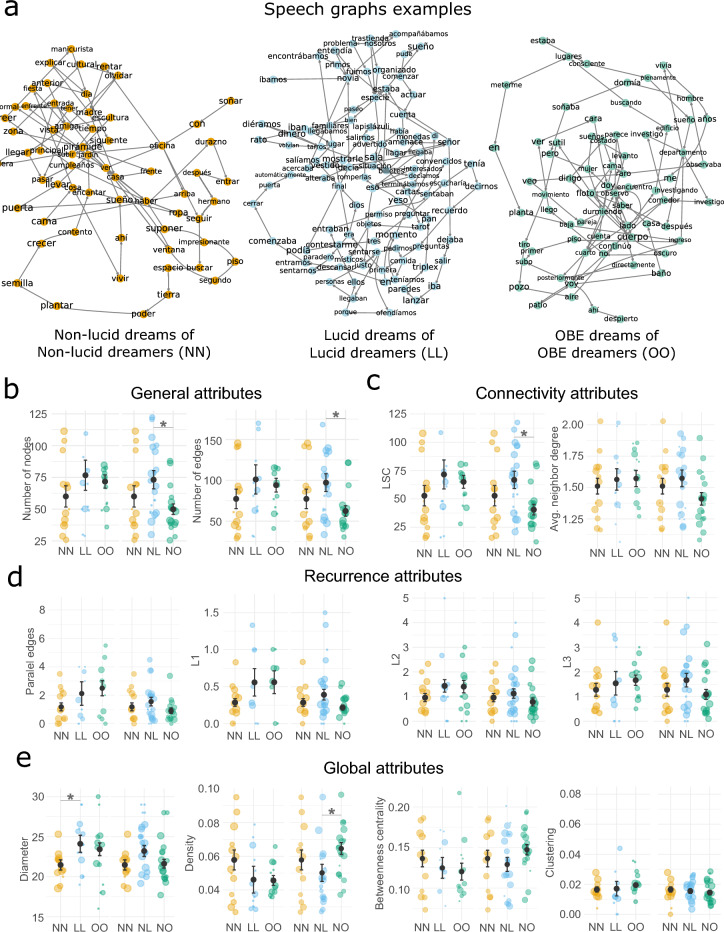
Network analysis of dream reports. (**a**) Examples of speech graphs constructed from NN, LL and OO lemmatized text, utilizing only nouns, verbs, and adjectives. (**b**) General attributes including number of nodes and edges. (**c**) Connectivity attributes, including the number of nodes on the largest strongly connected component (LSC) and average neighbor degree. (**d**) Recurrence attributes, including the number of parallel edges (PE) and the number of loops with one, two or three nodes (L1, L2, L3). (**e**) Global attributes, such as diameter, density, betweenness centrality and clustering. Asterisks show P-values for the F-test, *P < 0.05 (Supplementary Table 1).

A graph is a mathematical representation of a network with nodes linked by edges, formally defined as G = (N, E), with the set of nodes N = {w1, w2,…, wn} and the set of edges E = {(wi,wj)}^28,32^ (Fig. 1A). A speech graph represents the sequential relationship of spoken words in a verbal report, with each different word represented as a node, and the sequence between successive words represented as a directed edge^27,28,32^. We calculated a total of 12 speech graph attributes for each dream report, including general graph attributes (N, total number of nodes; E, total number of edges), recurrence (PE, parallel edges; L1, L2, and L3, loops of one; two and three nodes), connectivity (LSC, largest strongly connected component), and global attributes (average total degree, ATD; density; diameter; clustering, CC and average shortest path, ASP). ASP was calculated by determining the shortest path between every pair of nodes in the graph and then taking the average of all these shortest paths; (see^28^ for details). To address the variability in the number of dreams contributed by each subject, we proceeded to construct “average graphs” for each type of dream per dreamer. These “average graphs” were created by considering the individual dreams contributed by each subject and calculating the average of the aforementioned attributes from these individual graphs. This approach allowed us to obtain a more generalized representation of dream characteristics within each category.

### Co-occurrence networks

Networks were constructed using KH coder 3 software^33^ to visualize the relationship between the most frequent words in the text corpora of three types of reports: non-lucid dreams of non-lucid dreamers (NN), lucid dreams of lucid dreamers (LL), and OBEs of OBE dreamers (OO). We used the methods developed by Fruchterman and Reingold^34^ and Kamada and Kawai^35^ to determine word locations and ensure that the resulting network is easy to read. In this process, terms that frequently appear together were connected to illustrate the co-occurrence structure in the data. We constructed the co-occurrence network based on the adjacency of two word forms in sentence formation. The resulting networks provide a visual representation of the most frequent words and their relationships in the text corpora of three types of reports.

### Data exclusion

We removed outliers by comparing mean values for the number of nodes and edges per participant and dream type. We used the *Routliers* R package^36,37^ to remove data points of more than 3 MADs from the group median. This filtered out participants reporting exceptionally long or short dreams. After exclusion, we ended up with a total of 59 dreamers. Among them, there were 13 non-lucid dreamers, 24 lucid dreamers, and 22 OBE dreamers. Out of the lucid dreamers, 24 also contributed lucid dreams, while 15 of the OBE dreamers provided OBE experiences.

### Statistical analysis

**Figure 2:**
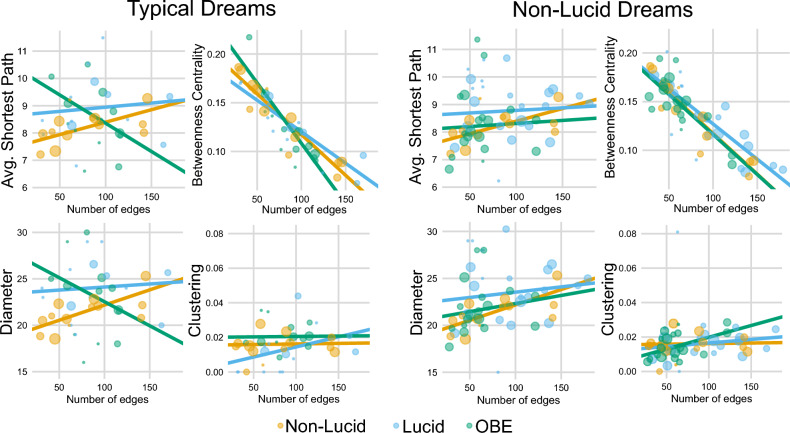
Correlation of network attributes. This figure displays the comparison between typical dreams (left; NN, LL and OO) and non-LDs (right; NN, NL and NO) through the fitting of linear models. Here we examine the relationship between the number of edges (nodes in text) with other network attributes such as diameter, clustering, average shortest path, and betweenness centrality.

We fitted weighted linear models to estimate the associations of the graph’s features with the type of dreamer for both, type of dreamer (non-LDs comparisons) and type of dreams (typical dreams comparisons; Fig. 2). We use weighted models instead of plain ones since there is an unbalanced number of dreams per dreamer. To account for this without overweighting the dreamers with more dreams, we weighted the data for the model fit using the logarithm of the number of dreams (log(N + 1)). We acknowledge that this collapsing process assumes the weighted mean effectively summarizes each dreamer’s behavior. We also fitted weighted linear models to estimate the associations of two of the graph’s features and the type of dreamer or the type of dream. For example, when modeling the Number of nodes vs the diameter for the non-LDs we fitted a full model with the Number of nodes as the dependent variable and the diameter and type of dream and its interaction as the independent variables. We use the *lm* function of base R to fit weighted linear models via the parameter *weights*. The parameter estimates and confidence intervals were calculated using the *broom* package in R^38^. We tested the fixed effects of both models with an F-test using the Anova function of the *car* R package^39^.

## Results

We first lemmatized and transformed each dream report into a graph and calculated its connectivity, recurrence and global attributes (Fig. 1, with graph examples shown in Fig. 1a). We found no significant differences between NN (non-LDs dreams of non-lucid dreamers), LL (LDs of lucid dreamers) and OO (OBEs of OBE dreamers) reports for any of the analyzed variables (Fig. 1b–e, Supplementary Table 1); except for diameter, with NNs having a smaller diameter than LLs, no other differences were observed between the groups (Fig. 1e, F(2, 37) = 3.32, P = 0.047, multiple comparisons: P_OOvsNN_ = 0.26, P_OOvsLL_ = 0.67, P_NNvsLL_ = 0.049). However, when comparing NN, NL (non-LDs of lucid dreamers), and NO (non-LDs of OBE dreamers) reports, we found significant differences in some of the attributes. Specifically, NO reports had a lower number of nodes than NL while no significant differences were found between other conditions (Fig. 1b, F(2, 52) = 3.95, P = 0.025, multiple comparisons: P_NOvsNN_ = 0.48, P_NOvsNL_ = 0.019, P_NNvsNL_ = 0.34). It is important to note that the total number of words did not significantly differ between conditions (NN: 217.3 ± 53.2 words, NL: 234.5 ± 44.4 words, NO: 169.7 ± 46.3 words; F = 2.15, P = 0.12).

Additionally, NO reports had a lower number of edges and largest strongly connected component (LSC) compared to NL reports. However, we did not find any differences between NN and NL or between NN and NO reports (Fig. 1b, Edges: F(2,52) = 3.87, P = 0.027, multiple comparisons: P_NOvsNN_ = 0.49, P_NOvsNL_ = 0.020, P_NNvsNL_ = 0.34; Fig. 1c, LSC: F(2,52) = 4.37, P = 0.017, multiple comparisons: P_NOvsNN_ = 0.39, P_NOvsNL_ = 0.012, P_NNvsNL_ = 0.34). We also found that NO graphs had a significantly higher density than the NL graphs. However, we did not find any differences between NN and NL or between NN and NO reports (Fig. 1e, F = 3.20, P = 0.048; multiple comparisons: P_NOvsNN_ = 0.55, P_NLvsNO_ = 0.038, P_NNvsNL_ = 0.41). No other differences were found for the average neighbor degree, parallel edges, loops of 1, 2 nor 3 nodes, betweenness centrality or clustering (Supplementary Table 1). These results showed that even though all reports had the same length, in NO reports, subjects used fewer distinct words to describe the experience than NL reports. This suggests that the disparity between NO and NL can be primarily attributed to the discrepancy in the number of nodes, as both edges and LSC are linearly dependent on this factor. In addition, the higher density observed in the NO reports, which is not linearly influenced by the number of nodes, suggests that despite having fewer overall edges, the fewer nodes in the graph are more densely connected. However, a broader range of connectivity measures, such as average neighbor degree or clustering did not align with this result.

Thus, due to the differences in the number of nodes across average graphs, we used linear models analysis to study if there were any variations in the relationships between node and edge attributes and other variables depending on the type of dream being considered (Fig. 2). Regarding the typical dreams (NN, LL and OO), we observed that there was a significant effect of the type of dream on the associations between ASP (a measure of network communication efficiency) and edges, but not between ASP and nodes (Fig. 2, edges: F(2,34) = 3.45, P = 0,043; nodes: F(2,34) = 2.27, P = 0.11). Furthermore, when observing the fitted slopes, we found that the ASP association for OO with nodes and edges was negative (edges: slope =  − 0.021, t(34) =  − 2.62, P = 0.012; nodes: slope =  − 0.024, t(34) =  − 2.13, P = 0.04), while the association for NN was positive, and the association for LL was not statistically significant (edges: NN slope = 0.009, t(34) = 1.98, P = 0.055; LL slope = 0.003, t(34) =  − 0.74, P = 0.46; nodes: NN slope = 0.014, t(34) = 2.11, P = 0.041; LL slope = 0.0098, t(34) =  − 0.35, P = 0.72). Moving on to the analysis of betweenness centrality, a measure that determines the centrality of a node based on the number of shortest paths passing through it, we also observed a significant effect of the dream type on the association with nodes and edges. We found significant associations between betweenness centrality and both nodes and edges (nodes: F(2,34) = 4.21, P = 0.023, Fig. 2, edges: F(2,34) = 3.87, P = 0.03). In addition, the association for OOs reports had a more pronounced negative association than NNs, while the association for LL was not statistically significant (Fig. 2, edges: NN slope =  − 0.0008, t(34) =  − 10.54, P < 0.0001; LL slope: − 0.0006, t(34) = 1.34, P = 0.18, OO slope: − 0.0012, t(34) =  − 2.06, P = 0.046; nodes: NN slope =  − 0.0012, t(34) =  − 10.37, P < 0.0001; LL slope: − 0.0009, t(34) = 1.13, P = 0.26, OO slope: − 0.0019, t(34) =  − 2.38, P = 0.022).

Regarding diameter, there was no significant effect of the type of dream for the association with nodes nor edges (nodes: F(2,34) = 1.80, P = 0.17, edges: F(2,34) = 3.04, P = 0.06, Fig. 2). Although, similar to ASP, the OOs showed a negative association with the number of nodes and edges while the association for NN was positive, and the association for LL was not statistically significant (nodes: OO slope =  − 0.054, t(34) =  − 1.87, P = 0.069; NN slope = 0.049, t(34) = 2.39, P = 0.022; LL slope = 0.023, t(34) =  − 0.68, P = 0.49; edges: OO slope =  − 0.051, t(34) =  − 2.416, P = 0.021; NN slope = 0.032, t(34) = 2.13, P = 0.027; LL slope = 0.059, t(34) =  − 1.04, P = 0.30, Fig. 2). It is important to highlight that the ASP and diameter are measures of network communication efficiency and size, respectively. Therefore, the increase in the number of nodes and/or edges in OBE dreams led to a decrease in diameter and ASP, in contrast to what was observed in NN and LL. Finally, concerning clustering, there was no significant effect of the dream type on the association with both nodes and edges (nodes: F(2,34) = 0.97, P = 0.38; edges: F(2,34) = 0.93, P = 0.40). Interestingly, we observed a different profile for the non-lucid dreams. We observed that for any of the attributes there was not a significant effect of the type of dream on nodes nor edges (Fig. 2, ASP, nodes: F(2,49) = 0.30, P = 0.73, edges: F(2,49) = 0.50, P = 0.61; between centrality, nodes: F(2,49) = 0.30, P = 0.73, edges: F(2,49) = 0.45, P = 0.63; diameter, nodes: F(2,49) = 0.29, P = 0.74, edges: F(2,49) = 0.48, P = 0.61; clustering, nodes: F(2,49) = 1.01, P = 0.37, edges: F(2,49) = 1.01, P = 0.37). These results suggest that there are distinct patterns in the network attributes and their associations with dream types, particularly in typical dreams (NN, LL, and OO). However, in non-lucid dreams, there were no significant effects observed on the associations between node and edge attributes and other variables. This lack of differences in non-lucid dreams implies that the variations found in typical dreams are not due to inherent differences between the three populations but rather stem from differences in the reported experiences themselves. This difference in findings between typical and non-lucid dreams indicates potential variations in the underlying cognitive processes involved in reporting these experiences.

**Figure 3:**
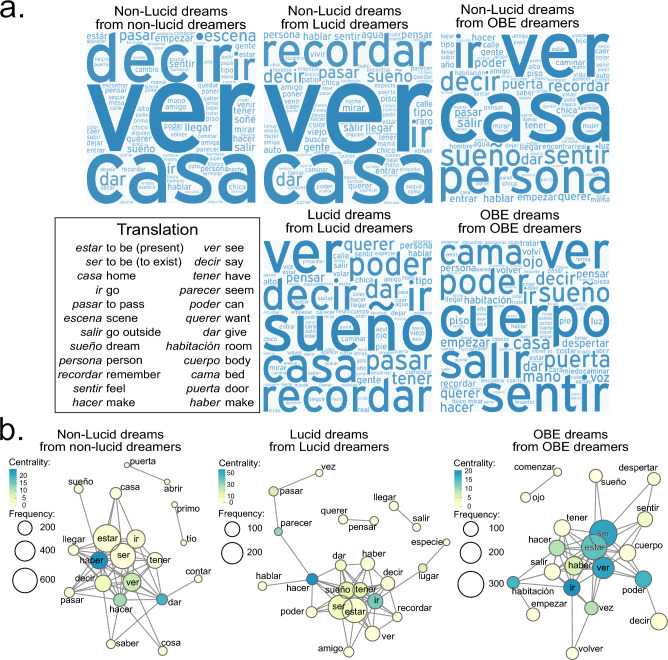
Dream report word clouds and co-occurrence networks. (**a**) Word clouds of the most frequent terms. This figure displays word clouds of the 100 most common terms in the lemmatized vocabulary of all dream reports analyzed in this study; all word clouds were generated using http://www.wordart.com. (**b**) Co-occurrence networks of dream reports. This figure presents co-occurrence networks constructed for the original text corpora of three types of typical dream reports. Co-occurrence refers to the adjacency of two words in sentence formation.

We further performed a qualitative analysis to identify common themes and patterns across different types of dreams (Fig. 3). The words “see” (*ver*) and “home” (*casa*) were most common across all dream reports, while “remember” (*recordar*) stood out in lucid dreamers’ reports and “person” (*persona*) in OBE dreamers’ reports. “Dream” (*sueño*) was prominent in both lucid and OBE dreams’ reports. When observing the reports of typical dreams, the frequency of the word “dream” (*sueño*) was the main difference between LL and OO, being higher in LL reports. This goes in line with previous studies showing that people who experienced OBEs, usually consider that it is not a dream but the veridical reality^9,13^. The terms “body” (*cuerpo*), “to leave” (*salir*), and “to feel” (*sentir*) were frequently used in OO reports but not in other types of dreams.

To visualize the general associations between the most frequent words, we constructed co-occurrence networks (Fig. 3; adjacency of two word forms in sentence formation) for the original text corpora for three types of reports (NN, LL and OO). For the NN reports, the most central words were “have” (*haber*), “give” (*dar*), “do” (*hacer*) and “see” (*ver*); for LL reports were “do” (*hacer*), “go” (*ir*), “dream” (*sueño*) and “have” (*haber*); and for OO reports were “to be” (*ser*), “see” (*ver*), “go” (*ir*), “room” (*habitación*), “can” (*poder*), “do” (*hacer*) and “being” (*estar*). Most notably, in the OO networks, among the most central words were “to be/to exist” (*ser*) and “to be/to be present” (*estar*), which were also the most frequent words in OO.

## Discussion

We found that OBE dreams are experiences different from lucid and non-lucid dreams. We observed that only for the OO reports, the higher the number of nodes (or edges), the less the diameter and the average shortest path. This suggests that as the length of the OBE reports increases, new words are not added to the narrative. On the contrary, OBE dreams present a more coherent and unified narrative, rather than one with many disparate or unrelated scenes or events. Thus, a dream with a more compact network structure would likely have fewer distinct elements or themes and more connections or relationships between those elements.

Furthermore, we found qualitative differences between OBE, LD and non-LD reports. We observed that there were words such as “see”, “home” and “say” that were common to all the dream reports while other words were more prominent in specific types of dreams. Notably, the term ‘remember’ appeared more prominently in both lucid and non-lucid dreams of the lucid dreamers (LL and NL). We suggest that this occurred because lucid dreamers often undergo training to enhance their ability to achieve lucidity during dreams. This training involves intending to remember to become lucid while they are asleep and actively thinking about ‘remembering the dream’. We consider that this pre-sleep intention to remember and the cognitive process of actively thinking about ‘remembering the dream’ can be easily incorporated into non-lucid dreams through ordinary memory processes, such as memory reactivation and integration that occur during sleep^40–42^ and can influence dream content^43^. Thus, the pre-sleep intention to remember and the cognitive focus on ‘remembering the dream’ during lucid dreaming training may contribute to the prominence of the term ‘remember’ in both lucid and non-lucid dreams of lucid dreamers.

Interestingly, the frequency of the term “dream” differed between LL and OO reports. This result supports the notion that individuals who experienced OBE often describe the episodes as highly vivid experiences, with the perceptual qualities resembling actual perception^9^. Additionally, it is common for individuals experiencing OBEs to believe that they are not dreaming^20^. It is worth noting that the most typical OBE dream involves the dreamers witnessing their own body lying on the bed, which leads them to perceive that they are actually departing from their physical form. This perception enhances the sense of realism and strengthens the conviction that they have genuinely left their body.

We also found that the words ‘body’, ‘to leave’, and ‘to feel’ were prominent in OBE dreams but absent in other dream types. Initially, one might attribute this to dream selection bias, given that OBE narratives require certain elements, including ‘to leave the body’. However, our co-occurrence network analysis revealed that ‘to leave’ and ‘body’ were not directly connected. Instead, connections were observed between “to leave” and other words such as “room”, “do”, and “go”, among others. Similarly, the word “body” was connected to words like “can”, “see”, “to be”, and “to feel”, among others. The presence of the words “to leave” and “body” cannot be solely attributed to selection bias. Thus, we suggest that OBE narratives utilize these expressions differently from lucid and non-lucid dreams, extending beyond the influence of classification bias.

All these words are directly related to the OBE episodes, where the dreamers commonly perceive as if they are leaving their physical body^9^. The co-occurrence networks for the dream reports showed that only for the OO networks the most frequent words coincided with the most central ones. This could be due to the increased recurrence of the OBE reports that is also evidenced in Fig. 2, showing that the higher the number of nodes (or edges), the smaller the diameter and the ASP.

Additionally, the betweenness centrality of the networks in OBE dreams showed a more pronounced negative trend in relation to the number of nodes compared to non-LDs and LDs. This finding suggests that OBE dreams have a more specialized network structure, characterized by certain nodes playing a more central role in the overall network. These nodes may have a more significant influence on the structure and content of the dream narrative.

In contrast, non-LDs and LDs displayed a less pronounced negative trend in relation to the number of nodes, indicating that these types of dreams may have a more diffuse network structure with fewer central nodes.

Regarding the graph attributes analysis, we did not observe any significant differences between typical dreams (NN, LL and OO, Fig. 1). It is worth noting that these attributes are typically used to compare more extreme cases such as Psychosis^28,29^ or Alzheimer’s disease based on verbal fluency^32^, rather than subtle differences between dream narratives. As such, correlations between certain attributes and the number of nodes were performed to examine whether any effects were more noticeable in these relationships rather than in means. However, graph attributes analysis reveal significant differences for non lucid dreams. That is, the NO displayed a lower number of nodes, edges, and nodes on the largest strongly connected component (LSC) compared to the NL. Additionally, the NO had fewer unique words but a higher density of connections than NL. One possible explanation for this, could be attributed to a greater focus of the OBE subjects on writing about conscious experiences rather than NO, as the total number of words between non-lucid dreams remain constant between dreamers (NN, NL, NO).

It is important to acknowledge the limitations of this study. Firstly, it is crucial to consider that self-reported dream reports are subjective measures that can be prone to inaccuracy and reliability issues. Dream reports can be influenced by interpretation and biases in memory recall, as well as the introduction of new elements upon recollection^2^. However, we sought to mitigate these limitations by collecting reports immediately upon awakening, thereby minimizing extended periods of wakefulness between the experience and the report. Lemmatization helps to reduce the variations in word forms by mapping them to their base or dictionary form. However, it is important to note that lemmatization alone does not fully address the issue of different forms of words, such as “he”, “she”, “woman”, “child”, and their synonyms, which may not be treated as the same word. As a result, there is a possibility that the recurrence of certain concepts or entities could be underestimated in this type of analysis. It is worth noting that educational levels of participants were not specifically controlled for, which could potentially influence their ability to express thoughts and experiences using richer vocabulary. However, despite this limitation, we employed a comparative approach and included non-lucid dreams as a control to observe specific differences related to dreamer types minimizing the impact of intrinsic differences among participant groups. Further research with larger samples is needed to gain a more comprehensive understanding of OBEs, lucid dreams, and non-lucid dreams.

In summary, graph analysis has shown promising potential in studying subjective experiences and can reveal subtle differences between LDs and OBEs. The findings suggest that OBEs may have a more tightly knit structure compared to LDs and non-LDs, which could provide valuable insights into the nature of these experiences.

### Supplementary Information

Supplementary Table 1.

## Data Availability

The datasets used and/or analyzed during the current study available from the corresponding author on reasonable request.

## References

[1] Siclari F, Larocque JJ, Postle BR, Tononi G (2013). Assessing sleep consciousness within subjects using a serial awakening paradigm. Front. Psychol..

[2] Rosen MG (2013). What I make up when I wake up: Anti-experience views and narrative fabrication of dreams. Front. Psychol..

[3] Holzinger B, Mayer L (2020). Lucid dreaming brain network based on Tholey’s 7 Klartraum criteria. Front. Psychol..

[4] LaBerge S (2010). Signal-verified lucid dreaming proves that REM sleep can support reflective consciousness. Int. J. Dream Res..

[5] LaBerge SP, Nagel LE, Dement WC, Zarcone VP (1981). Lucid dreaming verified by volitional communication during REM sleep. Percept. Mot. Skills.

[6] Stumbrys T, Erlacher D (2012). Lucid dreaming during NREM sleep: Two case reports. Int. J. Dream Res..

[7] Mota-Rolim SA (2015). Neurophysiological features of lucid dreaming during N1 and N2 sleep stages: Two case reports. Sleep Sci..

[8] Sheils D (1978). A cross-cultural study of beliefs in out-of-the-body experiences, waking and sleeping. JSPR.

[9] Blackmore SJ (1982). Out-of-body experiences, lucid dreams, and imagery: Two surveys. Am. J. Psychol..

[10] Irwin HJ (1988). Out-of-body experiences and attitudes to life and death. Am. J. Psychol..

[11] LaBerge S, Gackenbach J, LaBerge S (1988). The psychophysiology of lucid dreaming. Conscious Mind, Sleeping Brain.

[12] de Sá JSR, Mota-Rolim A (2016). Sleep paralysis in Brazilian folklore and other cultures: A brief review. Front. Psychol..

[13] Herrero NL, Gallo FT, Gasca-Rolín M, Gleiser PM, Forcato C (2022). Spontaneous and induced out-of-body experiences during sleep paralysis: Emotions, “AURA” recognition, and clinical implications. J. Sleep Res..

[14] Cheyne JA (2003). Sleep paralysis and the structure of waking-nightmare hallucinations. Dreaming.

[15] Hishikawa Y, Shimizu T (1995). Physiology of REM sleep, cataplexy, and sleep paralysis. Adv. Neurol..

[16] Blanke O (2005). Linking out-of-body experience and self processing to mental own-body imagery at the temporoparietal junction. J. Neurosci..

[17] Bünning S, Blanke O (2005). “The out-of-body experience: Precipitating factors and neural correlates”. The boundaries of consciousness: Neurobiology and neuropathology. Prog. Brain Res..

[18] LaBerge S (1985). Lucid Dreaming.

[19] Levitan L, LaBerge S, DeGracia DJ, Zimbardo PG (1999). Out-of-body experiences, dreams, and REM sleep. Sleep Hypn..

[20] LaBerge, S. & DeGracia, D. J. Varieties of lucid dreaming experience. In *Individual Differences in Conscious Experience* (ed. John Benjamins Publishing Company) 269–307 (Kunzendorf & Wallace, 2000).

[21] Dodet P, Chavez M, Leu-Semenescu S, Golmard JL, Arnulf I (2015). Lucid dreaming in narcolepsy. Sleep.

[22] Mainieri G, Maranci JB, Champetier P, Leu-Semenescu S, Gales A, Dodet P, Arnulf I (2021). Are sleep paralysis and false awakenings different from REM sleep and from lucid REM sleep? A spectral EEG analysis. J. Clin. Sleep Med..

[23] Voss U, Holzmann R, Tuin I, Hobson JA (2009). Lucid dreaming: A state of consciousness with features of both waking and non-lucid dreaming. Sleep.

[24] Terzaghi M, Ratti PL, Manni F, Manni R (2012). Sleep paralysis in narcolepsy: More than just a motor dissociative phenomenon?. Neurol. Sci..

[25] Siclari F (2017). The neural correlates of dreaming. Nat. Neurosci..

[26] Elce V, Handjaras G, Bernardi G (2021). The language of dreams: Application of linguistics-based approaches for the automated analysis of dream experiences. Clocks Sleep.

[27] Domhoff GW, Domhoff GW (1996). The Hall/Van de castle system of content analysis. Finding Meaning in Dreams. Emotions, Personality, and Psychotherapy.

[28] Mota NB (2012). Speech graphs provide a quantitative measure of thought disorder in psychosis. PLoS ONE.

[29] Mota NB, Furtado R, Maia PPC, Copelli M, Ribeiro S (2014). Graph analysis of dream reports is especially informative about psychosis. Sci. Rep..

[30] Martin JM (2020). Structural differences between REM and non-REM dream reports assessed by graph analysis. PLoS ONE.

[31] Gallo, F. T. *et al.* Lucid dreams and out-of-body experiences reports: Differences in emotional content, dream awareness, and dream control. 10.31234/osf.io/qf4z7 (2023).

[32] Bertola L (2014). Graph analysis of verbal fluency test discriminate between patients with Alzheimer’s disease, mild cognitive impairment and normal elderly controls. Front. Aging Neurosci..

[33] Higuchi K (2016). A two-step approach to quantitative content analysis: KH coder tutorial using anne of green gables (Part I). J. Asia Jpn. Res. Inst..

[34] Fruchterman TMJ, Reingold EM (1991). Graph drawing by force-directed placement. J. Softw..

[35] Kamada T, Kawai S (1988). A simple method for computing general position in displaying three-dimensional objects. Comput. Vis. Graph. Image Process..

[36] Leys C, Ley C, Klein O, Bernard P, Licata L (2013). Detecting outliers: Do not use standard deviation around the mean, use absolute deviation around the median. J. Exp. Soc. Psychol..

[37] Delacre, M. & Klein, O. *Routliers: Robust Outliers Detection. R Package Version 0.0.0.3*. https://CRAN.R-project.org/package=Routliers (2019).

[38] Robinson, D., Hayes, A. & Couch, S. *Broom: Convert Statistical Objects into Tidy Tibbles. R Package Version 0.7* 5 (2021).

[39] Fox J, Weisberg S (2019). An R Companion to Applied Regression.

[40] Malinowski JE, Horton CL (2014). Memory sources of dreams: The incorporation of autobiographical rather than episodic experiences. J. Sleep Res..

[41] Stickgold R, Malia A, Maguire D, Roddenberry D, O’Connor M (2000). Replaying the game: Hypnagogic images in normals and amnesics. Science.

[42] Rasch B, Born J (2013). About sleep’s role in memory. Physiol. Rev..

[43] Wamsley EJ, Stickgold R (2018). Dreaming of a learning task is associated with enhanced memory consolidation: Replication in an overnight sleep study. J. Sleep Res..

